# Increasing dopamine synthesis in nigrostriatal circuits increases phasic dopamine release and alters dorsal striatal connectivity: implications for schizophrenia

**DOI:** 10.1038/s41537-023-00397-2

**Published:** 2023-10-05

**Authors:** Sunil Srivastav, Xiaoying Cui, Roger Bitencourt Varela, James P. Kesby, Darryl Eyles

**Affiliations:** 1 Queensland Brain Institute, The University of Queensland, Brisbane, QLD Australia; 2 Queensland Centre for Mental Health Research, Brisbane, QLD Australia

**Keywords:** Schizophrenia, Psychology

## Abstract

One of the most robust neurochemical abnormalities reported in patients with schizophrenia is an increase in dopamine (DA) synthesis and release, restricted to the dorsal striatum (DS). This hyper functionality is strongly associated with psychotic symptoms and progresses in those who later transition to schizophrenia. To understand the implications of this progressive neurobiology on brain function, we have developed a model in rats which we refer to as EDiPs (Enhanced Dopamine in Prodromal schizophrenia). The EDiPs model features a virally mediated increase in dorsal striatal (DS) DA synthesis capacity across puberty and into adulthood. This protocol leads to progressive changes in behaviour and neurochemistry. Our aim in this study was to explore if increased DA synthesis capacity alters the physiology of DA release and DS connectivity. Using fast scan cyclic voltammetry to assess DA release we show that evoked/phasic DA release is increased in the DS of EDiPs rats, whereas tonic/background levels of DA remain unaffected. Using quantitative immunohistochemistry methods to quantify DS synaptic architecture we show a presynaptic marker for DA release sites (Bassoon) was elevated within TH axons specifically within the DS, consistent with the increased phasic DA release in this region. Alongside changes in DA systems, we also show increased density of vesicular glutamate transporter 1 (VGluT1) synapses in the EDiPs DS suggesting changes in cortical connectivity. Our data may prove relevant in understanding the long-term implications for DS function in response to the robust and prolonged increases in DA synthesis uptake and release reported in schizophrenia.

## Introduction

The dopamine (DA) hypothesis of schizophrenia, though now 60 years old^1^, remains fundamental to disease etiology, course and treatment^2^. The basis for this hypothesis has rested on the facts that DA releasing agents such as amphetamine produce psychotic symptoms in healthy individuals^3^ and DA D_2_ receptor antagonism remains necessary for antipsychotic efficacy^4^. Recent advances in positron emission tomography (PET) imaging techniques, and the development of radioligands selective for multiple aspects of DA signalling has allowed a more precise picture of subcortical DA dysfunction in patients. We now know that there is no replicable alteration in DA receptor or transporter density/function^5^. However, there appears to be a robust increase in DA uptake and synthesis capacity, as well as enhanced psychostimulant-induced DA release, in the dorsal striatum (DS) of people with schizophrenia^6^.

The cause of increased DA synthesis and release in those with schizophrenia is not well understood. DA neurons feature a range of functional properties, of which multiple could impact signalling and ultimately behaviour. For example, DA neurons show spontaneous activity and are randomly depolarised at a rate of 1–8 Hz^7–9^. This spontaneous DA release is known as tonic DA, which is involved in maintaining an interstitial constant low level of extracellular DA at resting potential. However, DA neurons also characteristically undergo burst firing, in response to various salient stimuli resulting in large transient increases in synaptic DA. This is referred to as phasic DA release^10^. In schizophrenia, it has been suggested that more DA neurons may be at a higher resting state, therefore stimuli that would otherwise be considered non-salient may also induce a phasic response leading to inappropriate actions^11,12^. However, it remains unknown whether the increased uptake and synthesis of DA reported in the DS of patients leads to an alteration in tonic or phasic DA, or both.

Architecturally, not all varicosities containing DA are functionally similar^13^. Approximately 30% contain a highly regulated set of proteins demarcating a site of high probability DA release^14^. These active zones are governed by specialised scaffolding proteins such as Bassoon, RIM-1, Elks, Piccolo, Munc-13 etc., which are densely concentrated at these zones and tether neurotransmitter rich vesicles with calcium channels, forming a site of high probability release^15,16^. It is plausible that an increase in the number of such sites may facilitate increased DA release in patients.

Other neurotransmitters such as glutamate are also implicated in schizophrenia^17,18^. Magnetic resonance spectroscopy (MRS) is typically used to assess glutamate and Gamma-aminobutyric acid (GABA) levels in brain. A recent meta-analysis of such studies in patients with schizophrenia has been conducted examining 14 brain regions. Surprisingly, an increase in Glx (the combination of glutamate and its precursor glutamine) in the basal ganglia was the only finding that survived correction for multiple comparisons^19^. The striatum integrates glutamatergic afferents coming from cortex and thalamus projecting onto the medium spiny neurons. These two afferents from the cortex and thalamus are selectively marked by their distinct subtypes of vesicular glutamate transporters VGluT1 and VGluT2, respectively^20^. It remains unknown whether the increased Glx (glutamate + glutamine) in the basal ganglia of people with schizophrenia is a consequence of enhanced cortical or thalamic glutamatergic activity, or both.

To investigate these clinical phenomena further we have constructed an animal model replicating a selective increase in DA synthesis in nigrostriatal circuits. This model is referred to as EDiPs (Enhanced Dopamine in Prodromal schizophrenia). By delivering the two rate-limiting enzymes in DA synthesis (GTP cyclohydrolase 1 and tyrosine hydroxylase) to the pars compact of the substantia nigra, we have produced a model producing a selective increase in DA release in the DS in response to low dose amphetamine, and impaired pre-pulse inhibition as observed in patients^21,22^. This model also reproduces an increase in Glx in the DS^21^. EDiPs is therefore ideally situated to clarify whether increased DA synthesis in the DS, as observed in patients, alters tonic/phasic nature of DA signalling and glutamatergic connectivity.

## Materials and methods

Please see Supplementary Material for additional methods

### Generation of unilateral EDiPs

Male Sprague–Dawley (SD) rats were acquired at age postnatal day (P) 28 from the Animal Resources Centre (ARC, Western Australia, Australia), and pair-housed with ad libitum food and water. The EDiPs construct was administered similarly to the previously reported procedure^21^ with the major variation that the active construct (huGCH-1+huTH) was administered to one substantia nigra and the control (huGCH-1) to the other (Supplementary Fig. S1). This unilateral procedure allowed each animal to act as its own control increasing statistical power and vastly reducing experimental animal numbers. P35 SD rats under isoflurane anaesthesia (4% induction and maintained at 1.5–2.5%), were injected with 1 µl of viral construct to the respective nigra (control or EDiPs) at coordinates (from bregma): A-P: −5.2 mm, M-L: ± 2.4 mm, D-V (from dura): −7.6 mm. Animals were then allowed to mature for a further 8 weeks before being subjected to Fast-Scan Cyclic Voltammetry (FSCV) for characterisation of phasic DA dynamics. Of the 15 rats used, only seven had successful signal detection bilaterally and are therefore the only animals reported here. All immunohistochemical data was based on brains from these same seven animals. For an examination of background or tonic DA, no net flux (NNF) microdialysis was performed on a separate cohort of 10 rats of the same age. Experiments were conducted with approval from the Animal Ethics Committee of The University of Queensland (QBI/392/19 and 2021/AE001004).

The current study uses a unilateral EDiPs model, thus it is important to consider the possibility of confounds from contra-lateral projections of DA neurons. In rodents DA fibres from the substantia nigra pars compacta, the ventral tegmental area (VTA) and posterior hypothalamic area have only 1–3% contralateral projections into the DS^23^. Thus, we consider the contribution of afferents from the contralateral EDiPs substantia nigra would be minimal.

### Determination of phasic dopamine using Fast Scan Cyclic Voltammetry (FSCV)

FSCV was used to determine phasic DA release within the dorsal striatum of unilateral EDiPs rats 8 weeks post viral delivery. DA release was evoked by electrically stimulating the medium forebrain bundle (MFB) containing ascending axons from the nigra that project to the DS. The MFB includes ascending dopaminergic projections from both VTA and SN (substantia nigra) diverging to various regions of forebrain including DS, NAc, amygdala, olfactory tubercle and prefrontal cortex^24–26^. For details on probe positions, FSCV settings and how DA concentrations were calculated see supplementary methods. An exponential one phase decay profile was also used to determine the half-life of evoked DA, as an indirect method to estimate density of DA transporters (DAT)^27–29^. Only data from animals, in which an intact DA like oxidation profile could be achieved simultaneously from both hemispheres are reported here (seven out of 15 animals). At the end of sampling, rats were euthanized using pentobarbitone sodium followed by cardiac perfusion with 4% paraformaldehyde solution before harvesting the brain for subsequent immunohistochemistry studies.

### Immunohistochemistry and Image analysis

From the animals in which there was a bilateral phasic DA release response, striatal brain blocks were paraffin fixed and sectioned at 10 µm. A single section containing both the dorsal striatum (DS) and Nucleus Accumbens (NAc) was selected corresponding to approximate bregma 0.96 mm in an adult rat brain^30^. For DS, a point, 1 mm ventral from the apex of the corpus callosum was chosen. From this ventral position we sampled tissue 1 mm medially and 1 mm laterally corresponding to the medial and lateral DS respectively. For the NAc a point 0.3 mm from the anterior commissure was sampled as NAc core and a separate point 0.3 mm medially outside of core was taken for NAc shell. As we found no difference between location chosen within the DS or NAc the data are presented as the mean for DS and NAc accordingly. These positions are depicted in Supplementary Fig. S2. All immunohistochemical and microscopy methods are detailed in supplementary methods.

Deconvoluted images were imported into Imaris (Bitplane, version 9.6) for augmentation. 3D cell-surfaces were created for TH positive fibres, VGLUT1 and VGLUT2 using the Imaris “create surface” module. The Imaris “spot” module was used to create “spots” for Bassoon and PSD95. When Bassoon “spots” were colocalised within TH “surfaces” this was considered a site of “high probability DA release”. A glutamatergic synapse was defined as the presence of a VGluT marker within 100 nm of the generic postsynaptic marker PSD95. This distance was chosen as it was reported to be the synaptic length between VGluT1 and PSD95 using these same immunohistochemical markers^31^. To calculate number of such “*synapses*” a filtration feature was used to pair PSD95 with VGlut1 or VGlut2. For image processing detail see supplementary methods.

### Determination of tonic or background DA using No Net Flux microdialysis

No net flux microdialysis was used to determine background DA levels in the DS as a measure of tonic DA. This method is generally considered superior to a single microdialysis measure as it is based on a series of DA concentrations dialysed in a random order across the brain to create a regression line of the DA concentration of the inflow vs outflow. For a detailed description of probe positions, experimental design and HPLC conditions to determine dialysate DA concentrations (see supplementary methods).

### Statistics

Statistical analyses were conducted using GraphPad Prism (version 9.41.1, GraphPad Software). Two tailed paired *t*-tests were used to compare tonic and phasic DA levels between control and EDiPs hemispheres within same animal. Repeat measures (RM) ANOVA was used to compare relative synaptic densities in the DS and NAc. Data obtained from both brain regions was corrected using Šídák’s multiple comparisons test. Normality test for Gaussian distribution was performed where *t*-test was used for analysis. All the data sets met criteria for normal distribution when analysed with both Shapiro–Wilk and Kolmogorov–Smirnov test. Correlation between phasic DA and Bassoon within TH was analysed with Pearson’s correlation. Data are expressed as mean ± SEM *p < 0.05.

## Results

### Phasic DA release is increased in the EDiPs DS

**Fig. 1:**
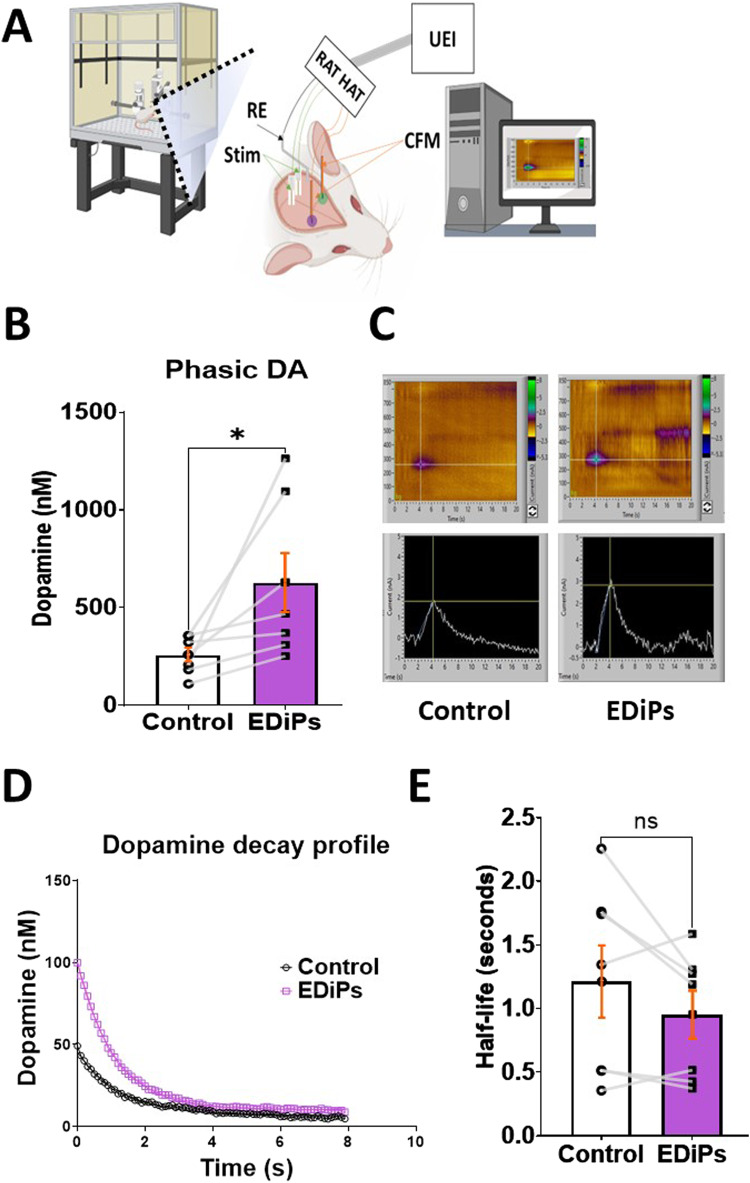
Determination of phasic DA release. **A** Simplified representation of fast scan cyclic voltammetry (FSCV) experiment set up. **B** DA concentrations showing increased stimulated release in the EDiPs when compared with the control hemisphere (*t*_(6)_ = 2.545, *p* < 0.05). **C** Pseudo colour plots showing strength of evoked DA release on stimulation. **D** DA decay profile in the DS after reaching peak in EDiPs and control hemispheres. **E** Half-life of phasic DA decay, paired for control and EDiPs hemispheres showed no changes (*t*_(6)_ = 1.610, *p* > 0.05). Data are presented as mean ± S.E.M. (*n* = 7 per group, *p < 0.05, paired t-test). CFM carbon fibre micro-electrode, Stim stimulating electrodes, RE reference electrode, UEI Universal Electrochemistry Instrument).

Using FSCV, evoked DA release in response to median forebrain bundle stimulation was recorded as a measure of phasic DA. Evoked DA release was significantly greater in the DS of the EDiPs compared to the control hemisphere (*t*_(6)_ = 2.545, *p* = 0.044) (Fig. 1B, C). In addition, the half-lives obtained from evoked DA decay profile showed no differences between control and EDiPs implying DA uptake was unchanged (*t*_(6)_ = 1.610, *p* = 0.16) (Fig. 1D, E).

### High probability release sites are increased in the EDiPs DS

**Fig. 2:**
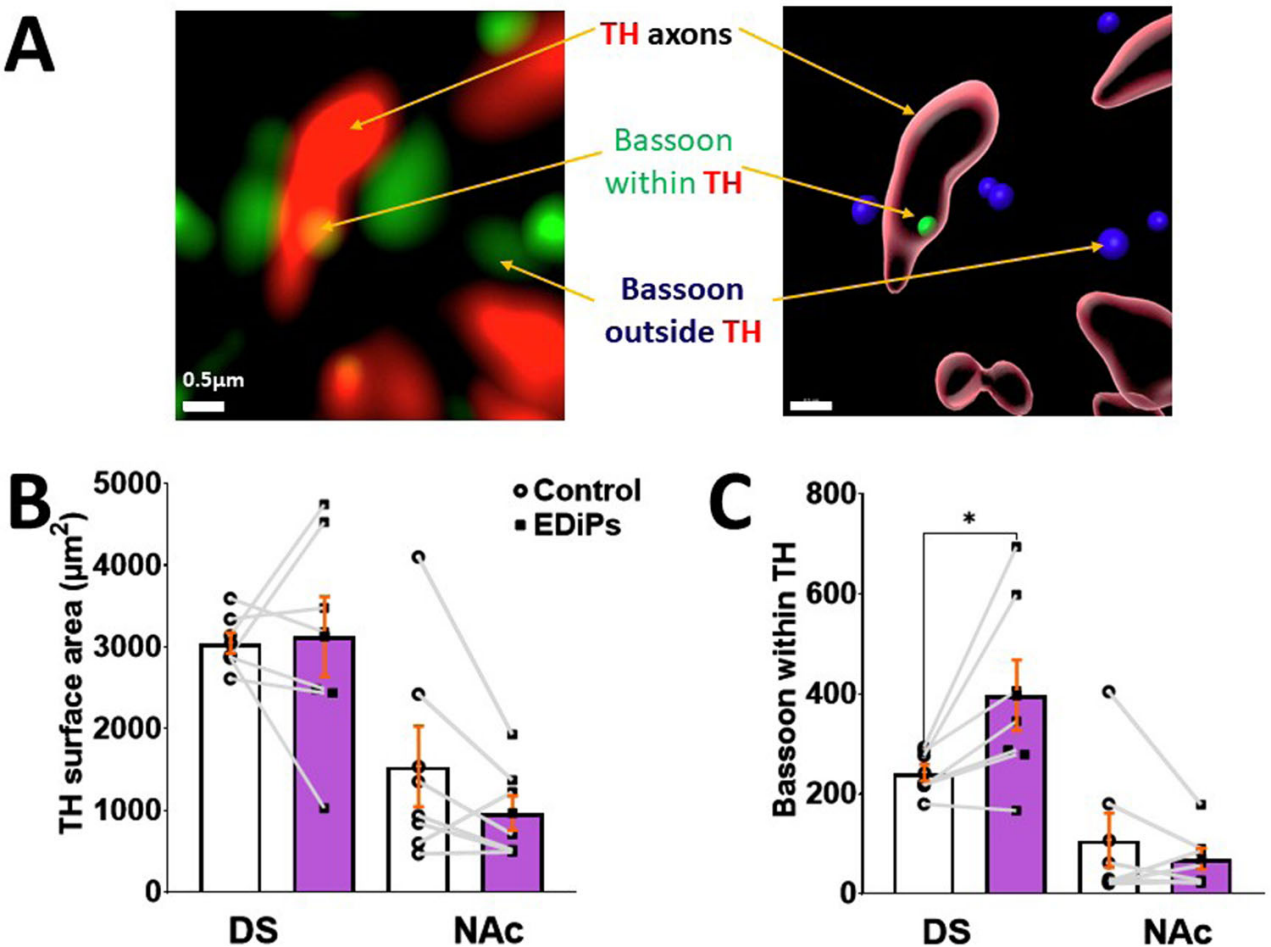
Effect of EDiPs construct on pre-synaptic architecture in DS DA terminals. **A** Raw microscopic image for TH and Bassoon converted into restructured image showing Bassoon within TH (green spot). **B** TH surface area in DS and NAc paired for hemisphere. **C** Number of Bassoon spots within TH surfaces in DS and NAc. (All data are presented as mean ± S.E.M. (*n* = 7 per group, *p < 0.05).

Increasing DA synthesis capacity in nigrostriatal circuits using the EDiPs construct was associated with an increase in evoked DA release in EDiPs DS terminals. We were interested in whether this was due to an increase in presynaptic release site density. Therefore, we quantified the number of high probability release sites using the presence of the presynaptic scaffolding protein Bassoon as a marker. When located inside a TH surface, this indicates a likely site of DA release (Fig. 2A). The TH surface area was unchanged in both the DS and NAc (Fig. 2B). RM ANOVA showed significant mixed effect of groups and brain region (*F*_(3,18)_ = 9.942), *p* = 0.0004) but after multiple comparisons with Šídák’s test there was no significant difference between the control and EDiPs hemispheres in both the DS (*p* = 0.98) and NAc (*p* = 0.45).

While comparing for number of Bassoon spots within TH, RM ANOVA analysis showed significant mixed effect of group and brain region (*F*_(3,18)_ = 11.85), *p* = 0.0002). And on Šídák’s multiple comparisons test the number of Bassoon spots (within TH surfaces) was significantly higher only in the DS (*p* = 0.04) of the EDiPs hemisphere compared with the control (Fig. 2C) indicating a greater number of high probability release sites. This was specific to the DS, as the number of Bassoon spots within TH surfaces of the NAc was unaltered (*p* = 0.80).

We further performed a correlation analysis to investigate if such an increase in high probability release sites were related to the elevation in phasic DA release (Supplementary Fig. S4). We found a weak but positive and significant correlation as an overall effect (*R*^2^ = 0.33, *p* = 0.03) between Bassoon within TH and phasic DA. However, we did not find any significant correlation when control (*R*^2^ = 0.29, *p* = 0.21) and EDiPs (*R*^2^ = 0.15, *p* = 0.39) hemispheres were analysed separately possibly due to reduced statistical power.

### Cortical but not thalamic connectivity is altered in the EDiPs DS

**Fig. 3:**
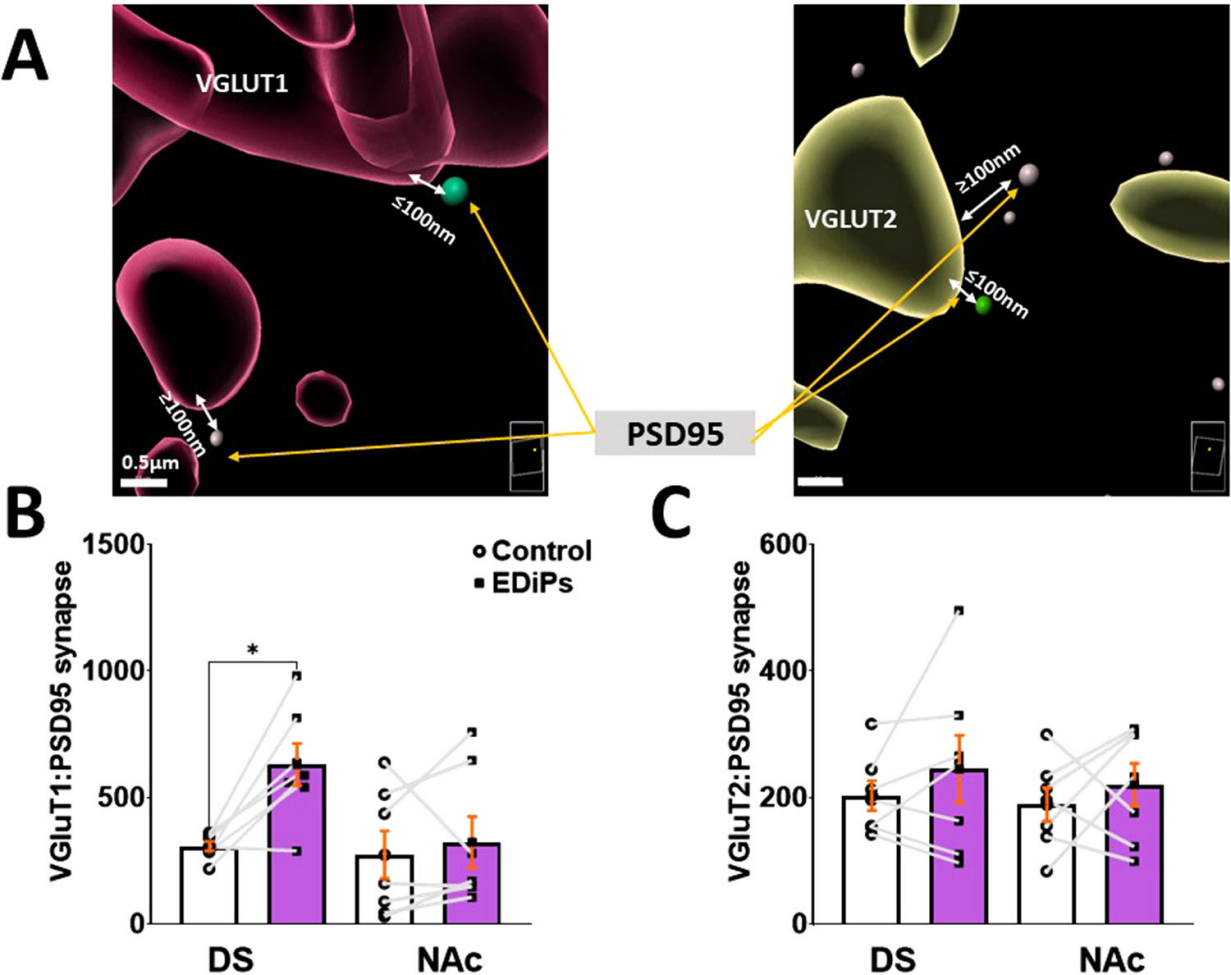
Effect of EDiPs construct on glutamatergic connectivity with the DS. **A** Restructured image for VGluT1:PSD95 synapse (green spot PSD < 100 nM from VGluT1 surface) and VGluT2:PSD95 synapse (green spot PSD < 100 nM from VGluT2 surface). **B** Number of VGluT1:PSD95 synapses in DS and NAc. **C** Number of VGluT2:PSD95 synapses in DS and NAc. All data are presented as mean ± S.E.M. (*n* = 7 per group, *p < 0.05).

Alongside dopaminergic inputs from the midbrain, the DS receives dense glutamatergic projections onto medium spiny neurons from both cortical and thalamic regions. The presence of different presynaptic transporters such as VGluT1 and VGluT2 can be used to identify those from cortical and thalamic sources, respectively^20^. We were interested in whether the changes in dopaminergic function and inputs to the DS led to compensatory changes in glutamatergic release, potentially related to the increased Glx present in the basal ganglia in patients with schizophrenia^32–34^. The number of cortical inputs (VGLuT1:PSD95 synapses) were elevated in the EDiPs DS hemisphere when compared to the control hemisphere NAc. RM ANOVA showed significant mixed effect of groups and brain region (*F*_(3,18)_ = 4.911), *p* = 0.01). After multiple comparisons with Šídák’s test the number of VGluT1:PSD95 synapses in the active EDiPs hemisphere was shown to be significantly higher than the control hemisphere but only in DS (*p* = 0.0137) being unchanged in NAc (*p* = 0.87) (Fig. 3B).

In contrast, the number of thalamic inputs (VGluT2:PSD95 synapses) were not different between hemispheres in either the DS or NAc (Fig. 3C). For VGluT2:PSD95 synapses, RM ANOVA showed no mixed effect of group and brain region (*F*_(3,18)_ = 0.60), *p* = 0.62), and no difference in (VGluT2:PSD95) densities between EDiPs and control hemispheres for both DS (*p* = 0.58) and NAc (*p* = 0.74).

### Regional comparisons between synaptic elements

The number of Bassoon spots within TH axons was higher in the DS than NAc but only in the active EDiPs hemisphere (*p* < 0.0001) being unchanged in the control (*p* = 0.08) (Supplementary Fig. S5A). We found no differences in VGluT1:PSD95 synapse number between DS and NAc in control (*p* = 0.94), but a significant elevation in the EDiPs hemisphere (*p* = 0.0188) (Supplementary Fig. S5B). There were no differences in VGluT2:PSD95 synapse number between DS and NAc in either control (*p* = 0.94) or EDiPs hemispheres (*p* = 0.82) (Supplementary Fig. S5C).

### Tonic/background DA is unaffected by the EDiPs construct

**Fig. 4:**
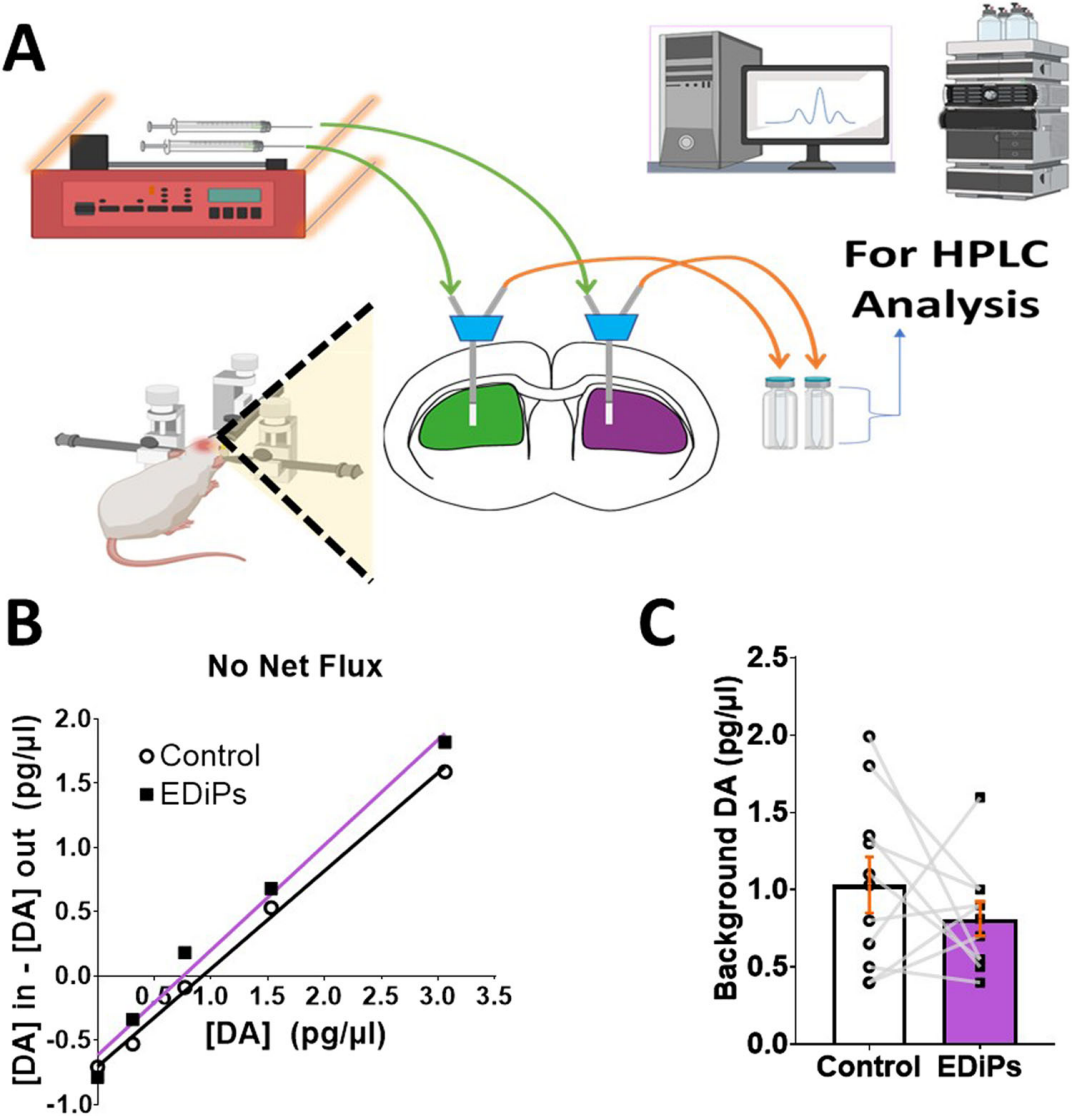
Determination of tonic or background DS DA levels. **A** Representative image showing general set up for no-net flux microdialysis experiment. **B** Representative no net flux microdialysis linear regression plot for one animal. **C** Background or tonic DA concentration paired for control and EDiPs hemispheres showing no effect (*t*_(9)_ = 0.9707; *p* > 0.05) and (**C**) Linear regression showing no-net flux profile. Data are presented as mean ± S.E.M. (*n* = 10 per group, *p > 0.05, paired *t*-test).

Given the alterations in phasic DA release and DA release site density in the DS of EDiPs hemispheres, we were interested in whether tonic DA levels were also impacted. Using No Net Flux microdialysis (Fig. 4A, B) to assess background/tonic DA, we show that basal extracellular levels of DA were unchanged between hemispheres (*t*_(9)_ = 0.9707; *p* = 0.36) (Fig. 4C).

## Discussion

The EDiPs model was constructed to mimic arguably the most robust neurochemical abnormality in schizophrenia, namely increased subcortical DA synthesis and release^6^. It replicates the anatomical selectivity of this abnormality in people with schizophrenia, restricting hyperdopaminergia to the DS. Moreover, its behavioural phenotypes of relevance to the positive symptoms of schizophrenia emerge progressively from adolescence to early adulthood, mimicking the temporal onset of symptoms in patients^35^. Together, these outcomes make this model ideal to study the prodromal onset of this disorder^21,22^. Now we extend the applicability of this model to understand how increasing DA synthesis selectively within the DS affects tonic/phasic DA release. We also have begun to explore the chronic impact of increased DA synthesis on other neurotransmitter systems in the DS, such as glutamate, which are also implicated in schizophrenia.

### Chronic increases in dopamine synthesis capacity increases evoked or phasic dopamine release: Possible presynaptic mechanism

We found evoked or phasic DA release was higher in the EDiPs hemisphere compared to its control. Phasic DA release is impacted by the density and functioning of DATs and and DA D2 autoreceptors. In particular DATs are more likely to impact phasic release, compared with tonic, as a fast means to re-uptake DA from burst release to bring down the synaptic DA levels to basal levels^36,37^. For example, DAT inhibitors such as methylenedioxypyrovalerone have been shown to increase phasic DA release^38^. Therefore, changes in the level or function of the DAT in EDiPs may account for these changes. The width of the evoked peak in FSCV indicates the time taken for re-uptake of DA, implying alterations to DAT function or density^39^. However, we did not find any changes to the width of the DA peak, indicated by the half-life of DA decay from the FSCV experiment in the current study. This would suggest DAT function in EDiPs is intact. Moreover, we have previously shown that mRNA levels of DAT in DS are similar between control and EDiPs^21^. In the absence of changes in the re-uptake of DA *after* a phasic response, changes in the presynaptic mechanisms that control phasic DA release may be causal.

An increase in evoked or phasic DA in the DS is consistent with an increase in the density of presynaptic release sites^40,41^. Bassoon-labelled sites, which were increased in TH terminals of the EDiPs hemisphere, undergo immediate depolarisation on MFB-stimulation^42^. Therefore, the increase in high probability DA release sites in EDiPs are a plausible mechanism for increased phasic DA release. Bassoon is one of multiple presynaptic scaffolding proteins that tether neurotransmitter laden vesicles with calcium channels indicating a high probability release site^43–45^. It has been estimated that 70% of DA release within the DS is non-synaptic^14^. This is referred to as volume transfer, where although the neurotransmitter released may still bind a postsynaptic DA receptor, this receptor is not in close apposition to the presynaptic site^46–48^. Thus, presynaptic release site number may provide a better picture of DA release capacity rather than DA synapse number.

Our findings are supported by studies in which selective DS knock-down of two other prominent presynaptic scaffolding proteins RIM and Munc-13 in DS DA neurons led to the opposite outcome, a reduction in DA release^41^. RIM expression is also highly correlated with Bassoon in TH positive striatal synaptosomes^14^. Excitatory postsynaptic currents have also been shown to be reduced in granule cell of cerebellar slices when Bassoon is reduced^49^.

### Alterations in dopamine synthesis capacity in the dorsal striatum induce reflexive changes in cortical glutamatergic synapses

Apart from DA, glutamate is another extensively studied neurotransmitter involved in schizophrenia. As previously mentioned, a very recent meta-analysis of magnetic resonance spectroscopy (MRS) studies in patients showed the only significant brain region affected (after multiple comparison corrections) was the basal ganglia in which Glx (the summation of brain glutamate and glutamine) was increased^19^. This suggests increased DS glutamate turnover in patients. Using MRS, we have previously examined baseline glutamine, glutamate and Glx levels in various brain regions of bilateral EDiPs animals and shown no alteration. However, upon stimulation with low dose amphetamine we showed a selective elevation in Glx selectively in the DS^21^. This indicates that local interactions between dopaminergic and glutamatergic systems in the DS are altered in this model. Given the close proximity between glutamatergic inputs to the DS and TH axons, DA released near these glutamatergic afferents may also affect their function^50^.

The DS receives major glutamatergic inputs from cortex and thalamus, for which VGluT1 and VGluT2 differentially mark their respective afferents in the DS^20^. Changes in glutamate transmission could be a functional outcome of altered levels of VGluT because its increase would result in more glutamate being packaged in vesicles potentially leading to increases in glutamate release^51^. So, to establish whether changes in glutamatergic innervation of the DS may underly altered Glx release in EDiPs^52^ (and by corollary, schizophrenia), we examined glutamatergic synaptic density in the DS. We observed increased levels of VGluT1:PSD95 synapses in the DS of the EDiPs hemisphere but not VGluT2:PSD95 synapses. This indicates a selective effect of increased DA release on cortical but not thalamic connectivity in the DS. L-DOPA, a precursor of DA synthesis, has been previously shown to restore cortico-striatal but not the thalamo-striatal glutamatergic synapses in a Parkinson’s disease model^53^. This was indicated by increased VGluT1^+^ synapses and unaltered VGluT2^+^ synapses after L-DOPA administered directly to the striatum. This supports the idea that increases in DA synthesis could selectively influence cortico-striatal glutamatergic afferents in the DS. Thus, it would appear that conditions that drive DA release within an environment primed to synthesise DA leads to increased glutamate turnover possibly as a cortical response to inappropriately heightened DA signalling.

Corticostriatal circuitry is heavily implicated in the psychotic and cognitive symptoms of schizophrenia^54,55^. With regards to reward circuitry, we have proposed that early subcortical dopaminergic alterations may precede more widespread cortical problems^55^. The data from EDiPs supports this premise, whereby subcortical dopamine disruptions lead to alterations in cortical connectivity, be they compensatory or a downstream consequence via other circuits. What this means for cognition in the EDiPs model remains to be seen. But work in mice has shown that sustained increases in the activity of dorsal striatal dopamine inputs can impair goal-directed behaviour^56^ and cognitive flexibility^57^. These impairments are reminiscent of those observed in early and persistent psychosis^58^. Of particular interest to the EDiPs model are mice lacking the DAT, which feature sustained increases in DA transmission in the striatum and hyperactivity^59^. Blocking glutamatergic transmission (via NMDA receptor blockade) in these mice exacerbates the behavioural phenotype, whereas increasing glutamatergic transmission (via AMPA agonists) attenuates the behavioural phenotype^59^. Consistent with this data are models of Parkinson’s disease where DA is *depleted* glutamatergic synapses on MSNs in the striatum are significantly *depleted*^60^. Taken together, the interactions of corticostriatal glutamatergic and nigrostriatal dopamine inputs to the striatum highlight how local compensation commonly occurs. We propose that the increases in glutamatergic synapses in the EDiPs model, are a compensatory response to decrease the pathological outcomes associated with dopaminergic hyperfunction.

### Tonic or background DA is unchanged in EDiPs

Here we show that experimentally increasing the capacity for DA synthesis in the nigrostriatal pathways leads to increased phasic responses but unchanged tonic DA. With increased capacity to synthesise DA one may expect that terminal content of DA may be similarly elevated. However, prior use of potassium chloride to release stored DA from terminals in bilateral EDiPs rats from a previous study showed no significant elevation above controls^21^. However, potassium chloride is likely to affect both the readily releasable DA pool (defined here as DA within Bassoon tethered vesicles) and the so-called reserve pool^61,62^. The reserve pool DA content vastly out numbers the releasable pool^63–65^ thus potentially obscuring any potassium chloride effect on the releasable pool. Another possibility may be that with increased synthesis capacity, DS DA terminals may just increase internal DA turnover to decrease any oxidative burden induced by DA production. However, we have previously reported baseline levels of DOPAC (3,4-dihydroxyphenylacetic acid) and HVA (Homovanillic acid), the major DA metabolites, are unchanged in EDiPs^22^ supporting our findings here of no changes in tonic DA. Therefore, it may be that compensatory mechanisms are able to maintain the level of tonic DA after EDiPs but are insufficient for phasic responses.

The regulation and modulation of tonic DA is primarily dependent on the density and functioning of DA D_2_ autoreceptors^36^. In our previous publication, we did not find any effect of quinpirole (a selective D2/3 receptor agonist) on locomotion at both low and high dose, where low doses of quinpirole preferentially target DA autoreceptors^66,67^. In addition, mRNA levels for the D2 short chain isoform indicating the DA autoreceptor were previously shown to be similar between control and EDiPs^21^. This is further indication of why tonic DA in EDiPs may be unaltered, as reported here.

One highly speculative mechanism for why phasic DA is increased in EDiPs, but not tonic DA, is that elevated levels of serotonin are impacting phasic dopamine release specifically. We have shown that baseline DS serotonin levels are elevated specifically in the DS (not PFC or Nac) of EDiPs animals^21^. Serotonin agonists and antagonists are known to reversibly regulate tonic DA release via the 5-HT_2C_ receptor with agonists decreasing dorsal striatal DA^68^ and antagonists increasing DA levels^68–73^. Thus, enhanced tonic serotonin in the DS of this model is likely to counter any increase in background or tonic DA levels resulting from increased DA synthesis. Interestingly when terminal release was assessed in bilateral EDiPs animals using a depolarising concentration of KCl, a reanalysis of previously published data from our group^21^ revealed the percent of 5-HT released relative to baseline was *decreased* again selectively in the DS of EDiPs animals (see Supplementary Fig. S3). This implies that under burst firing, serotonergic inhibition of DA release in the EDiPs DS may be diminished, allowing for increased phasic responses as seen here. Although highly speculative such a mechanism would be consistent with unchanged tonic but enhanced phasic DA release in the DS of EDiPs rats.

### Is phasic DA relevant to schizophrenia?

One long-standing hypothesis in respect to DA physiology in schizophrenia is that DA neurons may be more tonically active and perhaps able to respond to both appropriate and inappropriate stimuli to modulate the phasic response^10–12^. However more recently it has been argued that changes to phasic rather than tonic DA may be more consistent with the positive and negative symptoms of schizophrenia. Using a computation model (based on various learning and error equations), Maia and colleagues showed that alterations to phasic DA could possibly explain both positive and negative symptoms whereas changes to tonic DA could only explain positive symptoms^74^. These authors also made the point that with ablation of phasic DA, positive symptoms are likely to be improved, however reducing tonic DA may fail to do so.

### Implications of increased DA synthesis and release in the dorsal striatum

The DS consistently represents the locus for DA abnormalities in schizophrenia. Here we have developed the EDiPs model based on this underlying circuit abnormality in schizophrenia to understand the impact of such changes on this crucial brain region. We show increasing DA synthesis selectively in this circuit leads to an adaption of presynaptic release machinery likely facilitating DA release in response to stimulation. We also provide data indicating alterations to cortical glutamatergic terminals consistent with both previous data from our laboratory using this model and the latest meta-analytical data describing increased glutamate turnover in the basal ganglia of patients^19,21^. Other groups have shown that inducing striatal hyperdopaminergia by experimentally increasing DA receptor expression affects cognition and prefrontal cortical function suggesting so-called “bottom up” dopaminergic mechanisms can affect cortical function^75–78^. This is also supported, indirectly, by clinical data in those with early and persistent psychosis^55^. Our work complements these studies by inducing DS selective hyperdopaminergia in a manner more consistent with what has been reported in patients with schizophrenia. The impact of elevated DA in the DS on corticostriatal glutamatergic inputs is consistent with other preclinical models. But whether these reflexive changes in DS glutamatergic signalling leads to broader changes in cortical function over time is not known.

## Conclusions

**Fig. 5:**
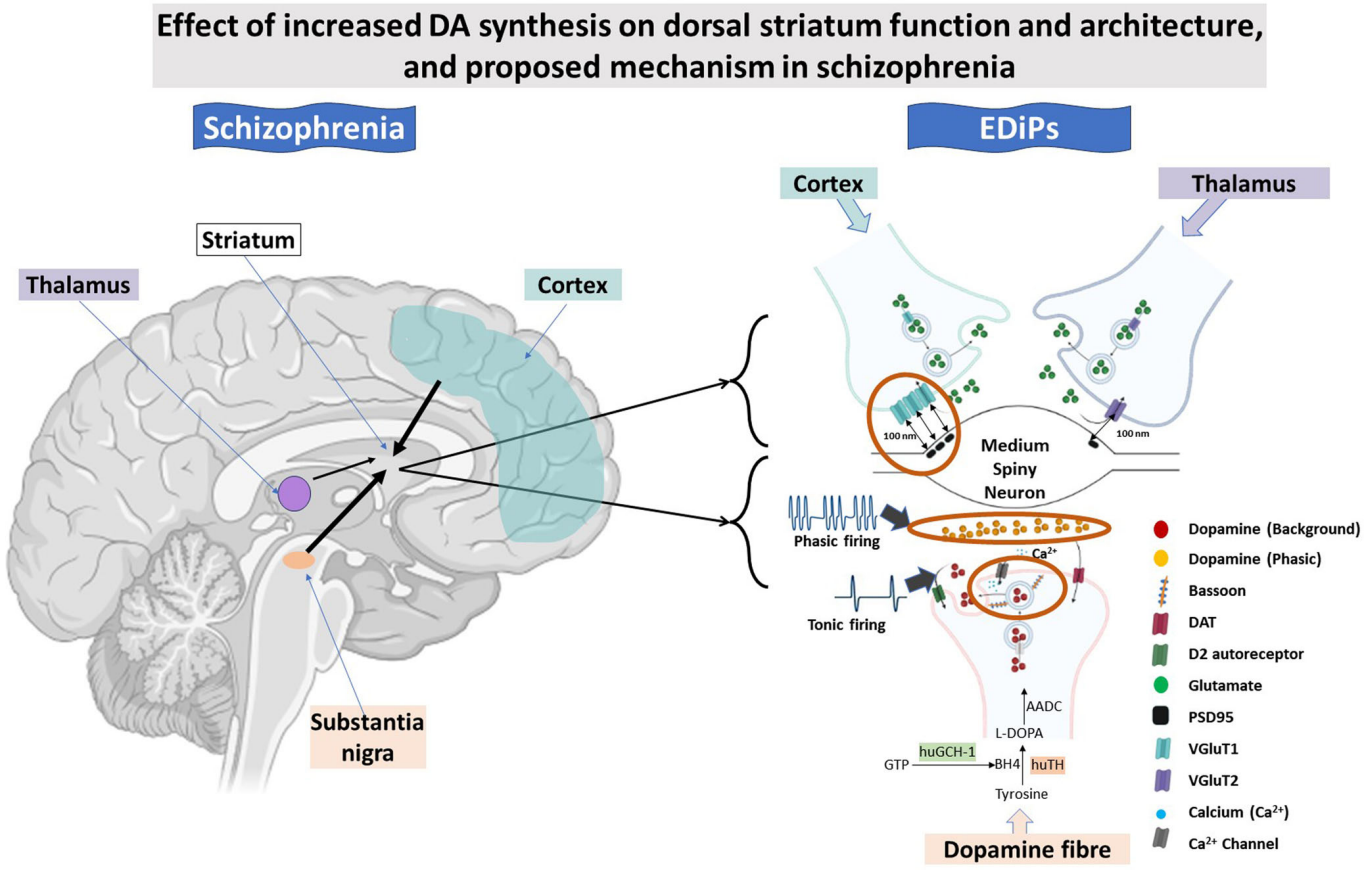
General effect of EDiPs on different aspects of DA release and synaptic architecture in the DS. The increased capacity to synthesise DA in the EDiPs hemisphere increases the capacity for presynaptic DA release. As we find no changes in DA re-uptake and unaltered tonic DA in EDiPs, the elevation in high probability release sites could be the causative mechanism for in the increased evoked or phasic DA release as observed in the EDiPs hemisphere. In addition, it also appears that such an increase in DA synthesis/release in DS of EDiPs could increase cortical glutamatergic connectivity. Whether such “bottom up” dopaminergic mechanisms have the same effect on “top down” cortical connectivity in schizophrenia remains unknown. The orange circles indicate major alterations observed in current study.

The EDiPs model continues to prove informative for understanding schizophrenia-relevant brain circuitry. By mimicking the dorsal striatal selective increase in DA synthesis capacity repeatedly observed in F^18^ DOPA PET studies in patients^5^ we have shown that this changes DS DA terminal architecture in a manner compatible with increased phasic DA release upon electrical stimulation. It is tempting to speculate that patients with schizophrenia may also have increased DA release within the DS in response to both salient and non-salient stimuli and the cortex responds by increasing glutamatergic synapse density in this region. Our study raises the possibility that both presynaptic DA terminals and cortical glutamatergic inputs are altered selectively in the DS in response to increased DS DA synthesis as observed in patients with schizophrenia. Our findings from the EDiPs model suggest a promising and possible mechanistic pathway through which subcortical pathology precedes, and even leads to widespread cortical dysfunction in the progression of schizophrenia^55^. The use of only male SD rats in current study, remains a limitation of this study. Our findings are summarised in Fig. 5.

### Supplementary information

SUPPLEMENTAL MATERIAL
