# Supplementary material for: Increasing dopamine synthesis in nigrostriatal circuits increases phasic dopamine release and alters dorsal striatal connectivity: implications for schizophrenia
Source: Schizophrenia (Heidelb). 2023 Oct 5;9(1):69. doi: 10.1038/s41537-023-00397-2 (PMC10556015; doi:10.1038/s41537-023-00397-2)
Supplement: Supplementary file 1 — SUPPLEMENTAL MATERIAL [file 41537_2023_397_MOESM1_ESM.docx]

**Supplementary Information**

**
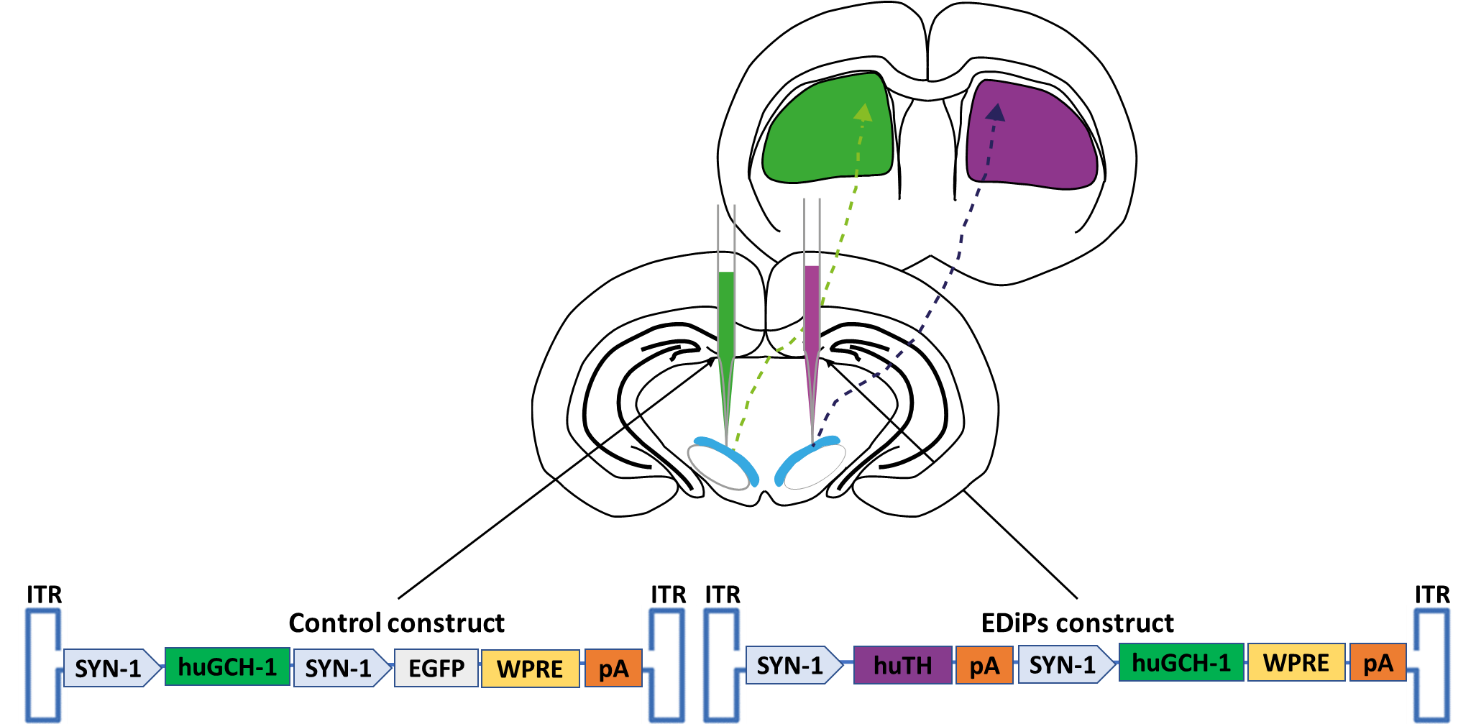
**

**Figure S1**. **Generation of the unilateral EDiPs model**. The virally packaged control construct was delivered into the nigra (bregma -5.2 mm) of the left hemisphere and the active EDiPs to the right nigra of same rat at five weeks of age. The dynamics of DA release was tested 8 weeks later when rats were 13-14 weeks of age. DA ITR: inverted terminal repeat, SYN-1: Synapsin 1, huGCH1: human GTP cyclohydrolase 1, pA: polyadenylation tail, huTH: human tyrosine hydroxylase, WPRE: Woodchuck Hepatitis Virus Post-Transcriptional Response Element.

**
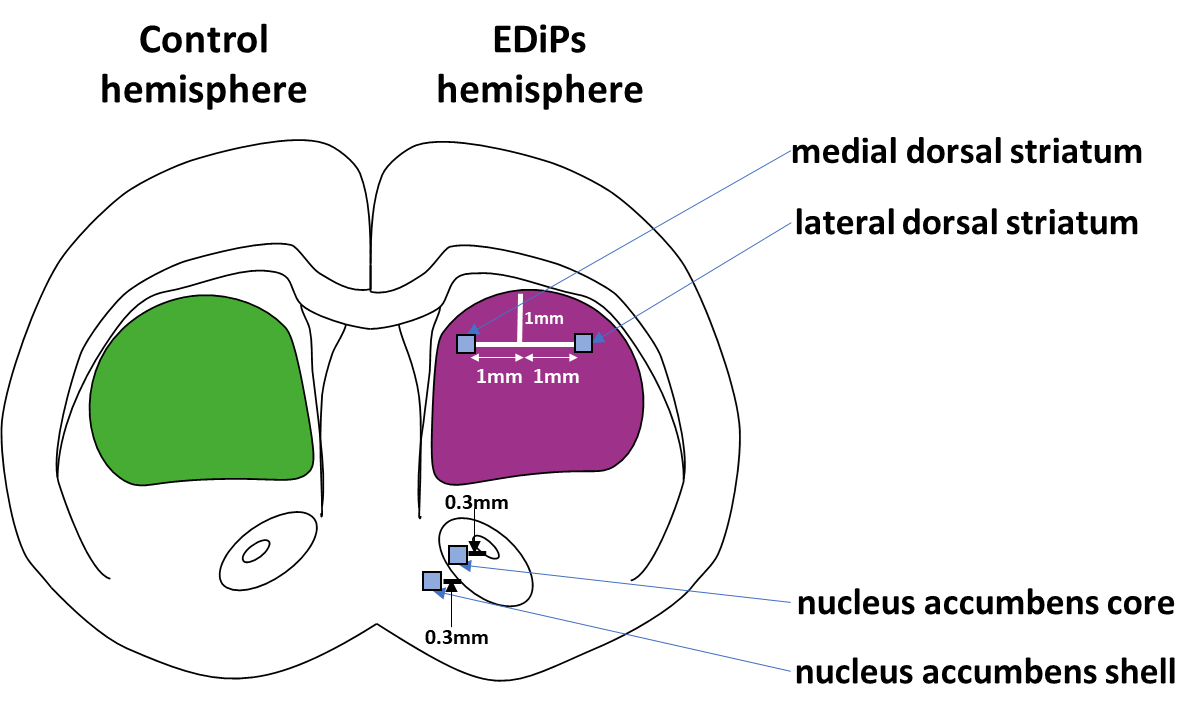
**

**Figure S2. Schematic representation of striatal brain regions selected for immunohistochemical studies**. These regions were selected based on dopaminergic projections of midbrain DA neurons, with ventral tegmental area projecting to nucleus accumbens (ventral striatum) and substantia nigra DA neurons projecting to the dorsal striatum [1,2] .


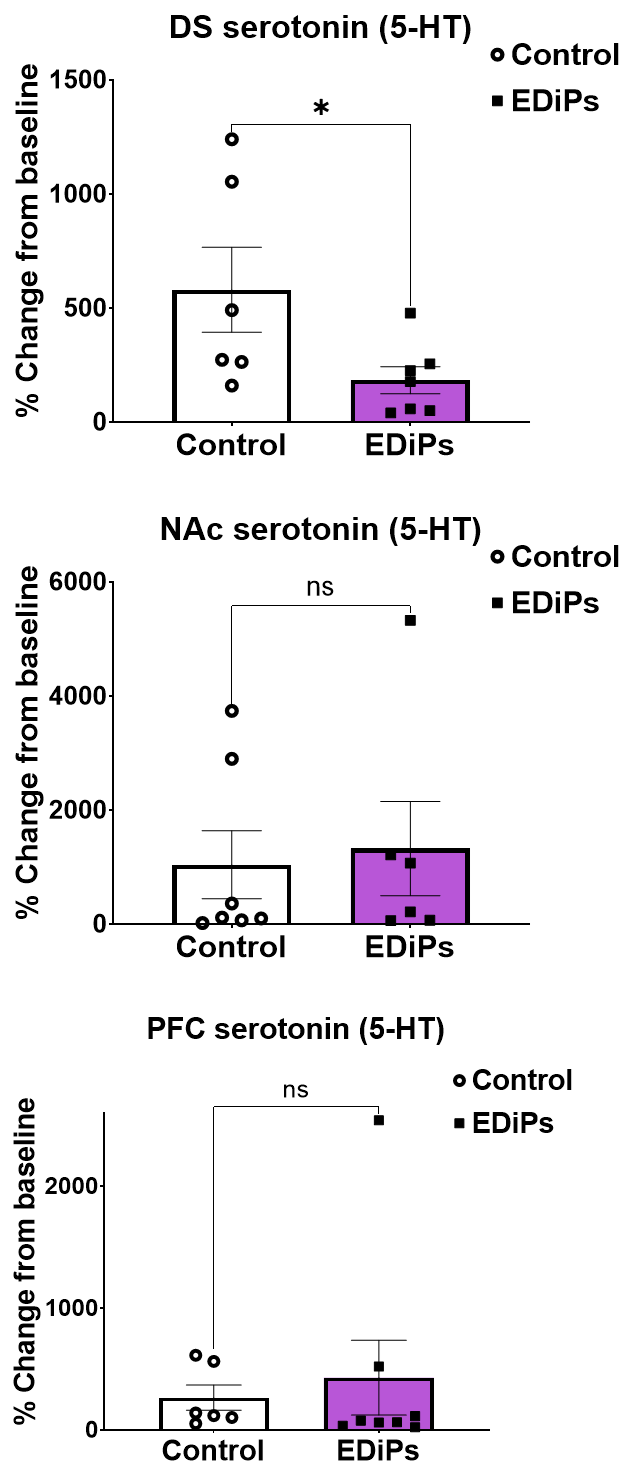


**Figure S3. Reanalysis of previously published data** in the bilateral EDiPs model. Percentage change from baseline for 5-HT in DS, NAc and pre-frontal cortex of bilateral EDiPs (reanalysis from previous data [3] after KCl challenge). All data are presented as mean ± S.E.M. (n = 6~7 (control), n = 6~8 (EDiPs), *p<0.05, two-tailed t-test, unpaired and non-parametric, Mann-Whitney test).

**
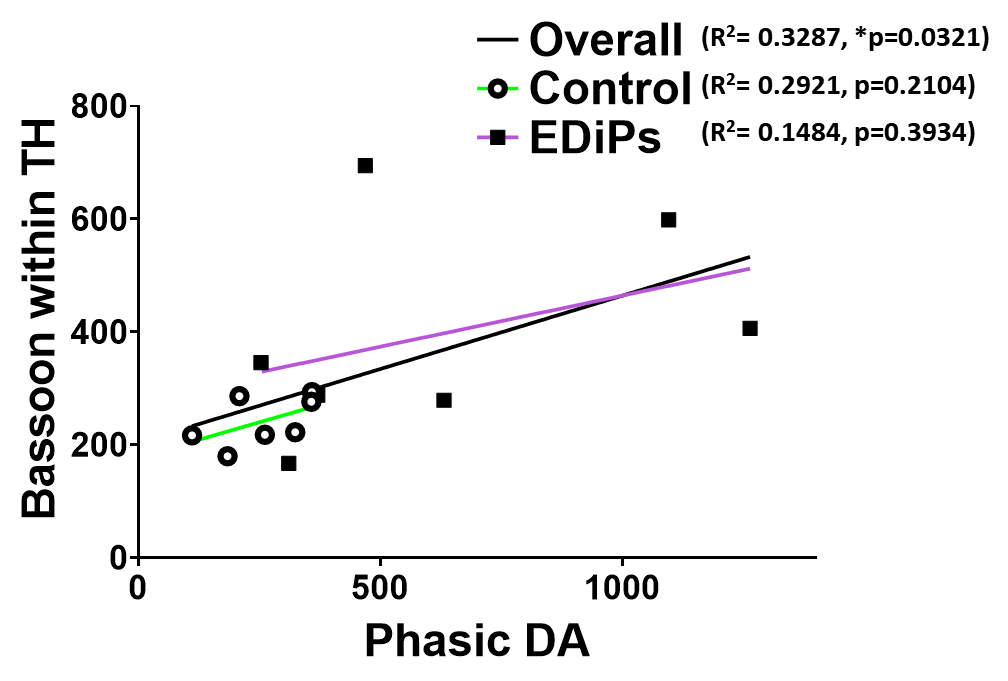
**

**Figure S4. Correlation between Bassoon spots within TH and phasic DA**. We observed an overall correlation between Bassoon with TH (as high probability release sites) and phasic DA. However, there was no correlations when analysed groupwise for control and EDiPs hemisphere possibly due to reduced statistical power. Spearman correlation, linear regression graph, n=7 (control), n=7 (EDiPs) and n=14 (Overall).

**
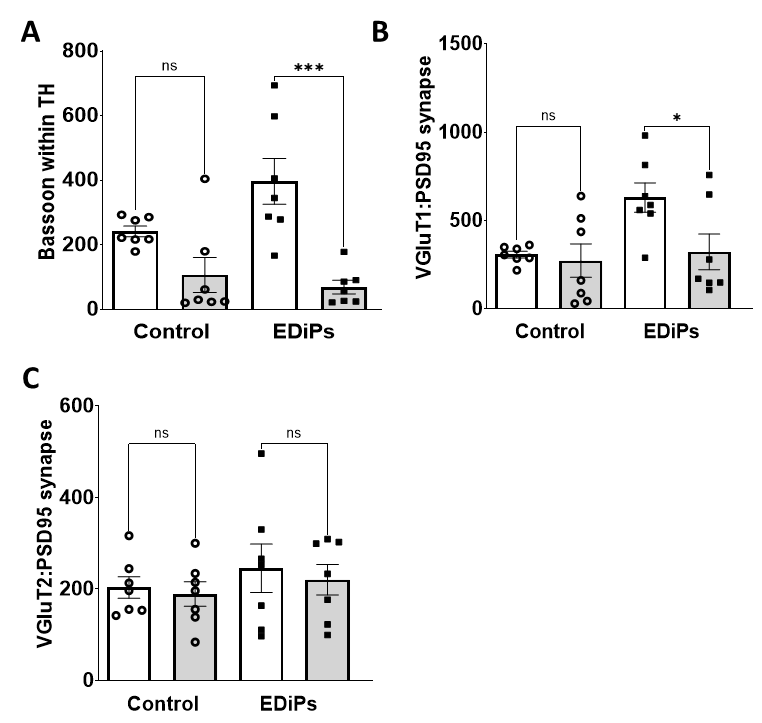
**

**Figure S5. Regional comparison between DS and NAc within the groups of control and EDiPs.**  (A) Bassoon spots within TH axons were significantly higher in the DS compared to NAc in active EDiPs hemispheres and unchanged in controls. (B) Similarly, VGluT1:PSD95 synapse number was significantly higher in DS of EDiPs compared to NAc and remained unchanged in controls, and (C) We did not any differences for VGluT2:PSD95 synapses between DS and NAc in either control or EDiPs hemispheres. All data are presented as mean ± S.E.M. (n = 7, RM ANOVA, *p<0.05, **p<0.01 and ***p<0.0001).

**Supplementary Table 1.**List of antibodies used in this study.

| S. No. | **Primary antibody** | | | | |
| --- | --- | --- | --- | --- | --- |
|  | Antibody | Host species | Titre | Catalogue # | Manufacturer |
| 1 | TH | Sheep | 1/200 | NB300-110 | Novusbio |
| 2 | Bassoon | Rabbit | 1/200 | 141003 | Synaptic systems |
| 3 | VGluT-1 | Guinea Pig | 1/1000 | AB5905 | Millipore |
| 4 | VGluT-2 | Rabbit | 1/500 | V 2514 | Sigma |
| 5 | PSD95 | Mouse | 1/200 | ab2723 | Abcam |

| **S. No.** | **Corresponding secondary antibody** | | | |
| --- | --- | --- | --- | --- |
|  | Antibody | Titre | Host species | Manufacturer |
|  | Alexa-555Anti-Sheep | 1/1000 | Donkey | ThermoFisher Scientific |
|  | Alexa-647Anti-Rabbit | 1/1000 | Donkey | ThermoFisher Scientific |
|  | Alexa-647Anti-Guinea Pig | 1/1000 | Donkey | ThermoFisher Scientific |
|  | Alexa-555 Anti-Rabbit | 1/1000 | Donkey | ThermoFisher Scientific |
|  | Alexa-488 Anti-Mouse | 1/1000 | Donkey | ThermoFisher Scientific |

**SUPPLEMENTARY MATERIALS AND METHODS**

**Fast Scan Cyclic Voltammetry**

FSCV surgeries were performed under non-recoverable anaesthesia using Urethane at dose of 1500mg/Kg (U2500-500G, Sigma). The rat was placed on heat pad on a stereotaxic frame (cat # 1430, KOPF) within a Faraday cage. The head of rat was shaved and locally prepared carbon fibre microelectrodes (CFMs) were positioned in the DS of both hemispheres at A-P: +1.2 mm, M-L: ±2.0 mm, D-V (from dura): −4.0~5.0 mm. Stimulating electrodes (MS303-13-B-SPC, Plastics One) were positioned within the medial forebrain bundle (MFB) of both hemispheres at A-P: −4.6 mm, M-L: ±1.2 mm, D-V (from dura): −8.0~9.0 mm, and the silver reference electrode (cat # 788000, A-M Systems) was positioned at A-P: −4.0 mm, M-L: 1.4 mm, D-V (from dura): −2.0 mm. All electrodes were connected through a head-stage into the UEI-HDCV (Universal Electrochemistry Instrument – High Definition Cyclic Voltammetry, system (CRiTCL, University of North Carolina) coupled with stimulating hardware (DS4 Bi-Phasic Current Stimulator, Digitimer Ltd.). A triangular waveform (-0.4 to 1.3V) at the rate of 400V/s and 10Hz frequency was applied at the CFM and stimulating electrodes had a biphasic pulse (frequency 60Hz, amplitude 2.0, pulse length 2 ms with number of pulses 120). The CFMs were later calibrated with method as described by Ramsson using an increasing range of DA concentrations [4]. The calibration curves thus obtained were used to convert DA oxidation response into DA concentrations.

## Immunohistochemistry methods and Microscopy

From the animals in which there was a bilateral phasic DA release response, striatal brain blocks were paraffin fixed and sectioned at 10 µm using Rotary Microtome (Leica RM2235, Leica Biosystems). Sections were deparaffinised, and antigen recovery was performed at 95°C for 10 minutes using decloaking chamber (NxGen, BioCare Medical) with 10 mM citrate buffer (pH 5.8) as antigen retrieval solution. This was followed by blocking solution containing bovine serum albumin for 4 hours. Then, the tissue sections were incubated with primary antibodies. After overnight incubation the sections were washed with PBS (phosphate buffer saline) and incubated with 4’,6-diamidino-2- phenylindole (DAPI, D8417, Sigma–Aldrich; 1:100,) along with species appropriate fluorophore-conjugated secondary antibodies for 4 hours. A full list of antibodies, titres and suppliers is provided in Supplementary Table 1. Sections were again washed with PBS and coverslipped using mounting medium (H-1000-10; Vectashield). Image capture was performed using a spinning disk confocal microscope (AxioObserverZ, Zeiss) equipped with a Yokogawa CSV-W1 unit. We used a Zeiss 100x (NA=1.4) objective lens for image acquisition on Slide 6.0 software (3i Intelligent Imaging Innovations) using a sCMOS Hamamatsu Flash 4.0 camera (2048 x 2048 pixels). Images were taken with a total Z stack of 2µm, acquired in 0.1µm steps (21 planes) using a piezo drive with an area of dimension 50µm × 50µm. The final voxel size was 62.5 x 62.5 x 100 µm.

**Image analysis techniques**

Images were restored using Huygens deconvolution software. Background was automatically assessed using a 0.7µm rolling ball approach. The applied deconvolution used the iterative CMLE (Classic Maximum Likelihood Estimation) algorithm with a threshold of 0.1 and 40 iterations. Deconvoluted images were imported into Imaris (Bitplane, version 9.6) for augmentation. The fluorescence threshold for determining objects was set manually (based on the individual background) to different antibodies. 3D cell-surfaces were created for TH positive fibres, VGLUT1 and VGLUT2 using the Imaris “create surface” module, and only puncta larger than three voxels (0.024 µm^3^) were selected. The Imaris “spot” module was used to create “spots” for Bassoon and PSD95. Bassoon spots were considered a site of “high probability release” when colocalised within TH “surfaces”. A glutamatergic synapse was defined as the presence of a VGluT marker within 100 nm of the generic postsynaptic marker PSD95. This distance was chosen as it was reported to be the synaptic length between VGluT1 and PSD95 using these same immunohistochemical markers [5]. To calculate number of such “*synapses*” a filtration feature was used to pair PSD95 with VGlut1 or VGlut2.

## No Net Flux microdialysis technique

Rats underwent isoflurane anaesthesia and two 3 mm dialysis probes (CMQ 12 Elite, Harvard Apparatus) were inserted into the DS of both hemispheres at A-P (from bregma): + 0.6 mm, M-L: − 2.6 mm & D-V (from dura): − 5.5 mm. Probes were infused with artificial Cerebral Spinal Fluid (aCSF) containing 154.1 mM Cl, 147 mM Na^+^, 2.7 mM K+, 1 mM Mg^2+^, and 1.2 mM Ca^2+^ (pH approximately 7.4) at 1 μl/min. Following a lengthy baseline (90 minutes of six × 15 minute bins) to allow extracellular DA levels to normalise after probe insertion, four separate aCSF solutions at different DA concentrations at 2 nM, 5 nM, 10 nM & 20 nM in random order were infused. Three dialysate samples were collected in 15 min bins for each concentration. Of six bins collected for baseline last five bins (corresponds to 0 nM for no-net flux) and all three bins from each DA concentration (2 nM, 5 nM, 10 nM & 20 nM) were used for analysis. Dialysates were analysed by high-performance liquid chromatography (HPLC) (Agilent Technologies, Inc., CA, USA). Mobile phase containing 50 mM Citric Acid.H_2_O, 25 mM NaH_2_PO_4,_ 1 mM EDTA, 3.2 mM octane sulfonic acid in 10% acetonitrile; pH adjusted to 5.00 at a flow rate of 0.6 ml/minute using a C-18 column (186002543, SunFire) and an electrochemical detector (Coulochem III; ESA Laboratories, Inc., MA, USA) coupled to an analytic cell (Model 5014B; ESA Laboratories, Inc., MA, USA) to detect DA was employed.

No-net flux is obtained after difference between concentrations of inflow DA perfusates [DA]_in_ and concentration of outflow dialysate [DA]_out_ was plotted against [DA]_in_ as a linear regression graph for each animal. The x – intercept of thus obtained line then indicates a more reliable estimate of baseline DA is the no-net flux DA.

**References**

1 Beckstead RM, Domesick VB, Nauta WJ. Efferent connections of the substantia nigra and ventral tegmental area in the rat. Brain Res. 1979;175(2):191-217.

2 Duzel E, Bunzeck N, Guitart-Masip M, Wittmann B, Schott BH, Tobler PN. Functional imaging of the human dopaminergic midbrain. Trends Neurosci. 2009;32(6):321-8.

3 Petty A, Cui X, Tesiram Y, Kirik D, Howes O, Eyles D. Enhanced Dopamine in Prodromal Schizophrenia (EDiPS): a new animal model of relevance to schizophrenia. NPJ Schizophr. 2019;5(1):6.

4 Ramsson ES. A pipette-based calibration system for fast-scan cyclic voltammetry with fast response times. Biotechniques. 2016;61(5):269-71.

5 Hruska M, Henderson N, Le Marchand SJ, Jafri H, Dalva MB. Synaptic nanomodules underlie the organization and plasticity of spine synapses. Nat Neurosci. 2018;21(5):671-82.
